# Supplementary figures and images for: Predicting central cervical lymph node metastasis in papillary thyroid microcarcinoma using deep learning (part 2 of 2)
Source: PeerJ. 2024 Mar 29;12:e16952. doi: 10.7717/peerj.16952 (PMC10984175; doi:10.7717/peerj.16952)

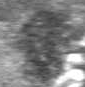

Supplement: Supplemental Information 2 — The raw data shows the demographics and clinicopathological features of all patient which were used to statistical analysis and built DL model. [file peerj-12-16952-s002.zip › Submited_Images/00132/11.tif]

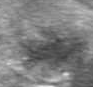

Supplement: Supplemental Information 2 — The raw data shows the demographics and clinicopathological features of all patient which were used to statistical analysis and built DL model. [file peerj-12-16952-s002.zip › Submited_Images/00133/22.tif]

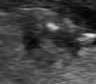

Supplement: Supplemental Information 2 — The raw data shows the demographics and clinicopathological features of all patient which were used to statistical analysis and built DL model. [file peerj-12-16952-s002.zip › Submited_Images/00134/33.tif]

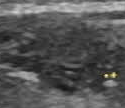

Supplement: Supplemental Information 2 — The raw data shows the demographics and clinicopathological features of all patient which were used to statistical analysis and built DL model. [file peerj-12-16952-s002.zip › Submited_Images/00135/11.tif]

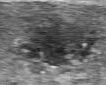

Supplement: Supplemental Information 2 — The raw data shows the demographics and clinicopathological features of all patient which were used to statistical analysis and built DL model. [file peerj-12-16952-s002.zip › Submited_Images/00139/44.tif]

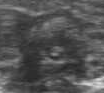

Supplement: Supplemental Information 2 — The raw data shows the demographics and clinicopathological features of all patient which were used to statistical analysis and built DL model. [file peerj-12-16952-s002.zip › Submited_Images/00140/22.tif]

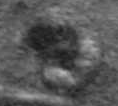

Supplement: Supplemental Information 2 — The raw data shows the demographics and clinicopathological features of all patient which were used to statistical analysis and built DL model. [file peerj-12-16952-s002.zip › Submited_Images/00141/22.tif]

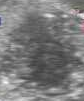

Supplement: Supplemental Information 2 — The raw data shows the demographics and clinicopathological features of all patient which were used to statistical analysis and built DL model. [file peerj-12-16952-s002.zip › Submited_Images/00142/22.tif]

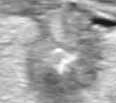

Supplement: Supplemental Information 2 — The raw data shows the demographics and clinicopathological features of all patient which were used to statistical analysis and built DL model. [file peerj-12-16952-s002.zip › Submited_Images/00143/22.tif]

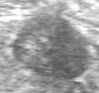

Supplement: Supplemental Information 2 — The raw data shows the demographics and clinicopathological features of all patient which were used to statistical analysis and built DL model. [file peerj-12-16952-s002.zip › Submited_Images/00144/22.tif]

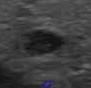

Supplement: Supplemental Information 2 — The raw data shows the demographics and clinicopathological features of all patient which were used to statistical analysis and built DL model. [file peerj-12-16952-s002.zip › Submited_Images/00145/11.tif]

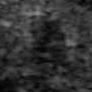

Supplement: Supplemental Information 2 — The raw data shows the demographics and clinicopathological features of all patient which were used to statistical analysis and built DL model. [file peerj-12-16952-s002.zip › Submited_Images/00146/77.tif]

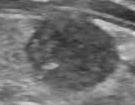

Supplement: Supplemental Information 2 — The raw data shows the demographics and clinicopathological features of all patient which were used to statistical analysis and built DL model. [file peerj-12-16952-s002.zip › Submited_Images/00148/11.tif]

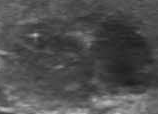

Supplement: Supplemental Information 2 — The raw data shows the demographics and clinicopathological features of all patient which were used to statistical analysis and built DL model. [file peerj-12-16952-s002.zip › Submited_Images/00149/44.tif]

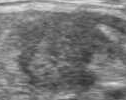

Supplement: Supplemental Information 2 — The raw data shows the demographics and clinicopathological features of all patient which were used to statistical analysis and built DL model. [file peerj-12-16952-s002.zip › Submited_Images/00151/11.tif]

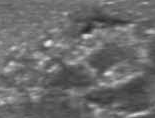

Supplement: Supplemental Information 2 — The raw data shows the demographics and clinicopathological features of all patient which were used to statistical analysis and built DL model. [file peerj-12-16952-s002.zip › Submited_Images/00152/11.tif]

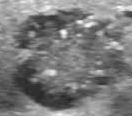

Supplement: Supplemental Information 2 — The raw data shows the demographics and clinicopathological features of all patient which were used to statistical analysis and built DL model. [file peerj-12-16952-s002.zip › Submited_Images/00153/11.tif]

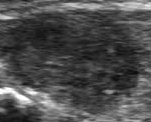

Supplement: Supplemental Information 2 — The raw data shows the demographics and clinicopathological features of all patient which were used to statistical analysis and built DL model. [file peerj-12-16952-s002.zip › Submited_Images/00154/22.tif]

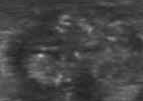

Supplement: Supplemental Information 2 — The raw data shows the demographics and clinicopathological features of all patient which were used to statistical analysis and built DL model. [file peerj-12-16952-s002.zip › Submited_Images/00155/22.tif]

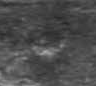

Supplement: Supplemental Information 2 — The raw data shows the demographics and clinicopathological features of all patient which were used to statistical analysis and built DL model. [file peerj-12-16952-s002.zip › Submited_Images/00156/22.tif]

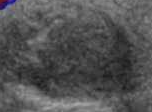

Supplement: Supplemental Information 2 — The raw data shows the demographics and clinicopathological features of all patient which were used to statistical analysis and built DL model. [file peerj-12-16952-s002.zip › Submited_Images/00157/44.tif]

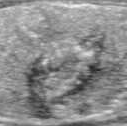

Supplement: Supplemental Information 2 — The raw data shows the demographics and clinicopathological features of all patient which were used to statistical analysis and built DL model. [file peerj-12-16952-s002.zip › Submited_Images/00158/44.tif]

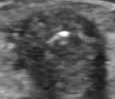

Supplement: Supplemental Information 2 — The raw data shows the demographics and clinicopathological features of all patient which were used to statistical analysis and built DL model. [file peerj-12-16952-s002.zip › Submited_Images/00159/11.tif]

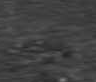

Supplement: Supplemental Information 2 — The raw data shows the demographics and clinicopathological features of all patient which were used to statistical analysis and built DL model. [file peerj-12-16952-s002.zip › Submited_Images/00160/33.tif]

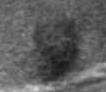

Supplement: Supplemental Information 2 — The raw data shows the demographics and clinicopathological features of all patient which were used to statistical analysis and built DL model. [file peerj-12-16952-s002.zip › Submited_Images/00163/33.tif]

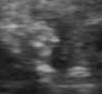

Supplement: Supplemental Information 2 — The raw data shows the demographics and clinicopathological features of all patient which were used to statistical analysis and built DL model. [file peerj-12-16952-s002.zip › Submited_Images/00165/22.tif]

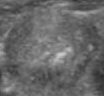

Supplement: Supplemental Information 2 — The raw data shows the demographics and clinicopathological features of all patient which were used to statistical analysis and built DL model. [file peerj-12-16952-s002.zip › Submited_Images/00166/44.tif]

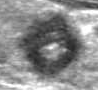

Supplement: Supplemental Information 2 — The raw data shows the demographics and clinicopathological features of all patient which were used to statistical analysis and built DL model. [file peerj-12-16952-s002.zip › Submited_Images/00168/11.tif]

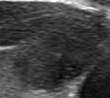

Supplement: Supplemental Information 2 — The raw data shows the demographics and clinicopathological features of all patient which were used to statistical analysis and built DL model. [file peerj-12-16952-s002.zip › Submited_Images/00169/11.tif]

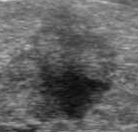

Supplement: Supplemental Information 2 — The raw data shows the demographics and clinicopathological features of all patient which were used to statistical analysis and built DL model. [file peerj-12-16952-s002.zip › Submited_Images/00170/44.tif]

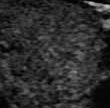

Supplement: Supplemental Information 2 — The raw data shows the demographics and clinicopathological features of all patient which were used to statistical analysis and built DL model. [file peerj-12-16952-s002.zip › Submited_Images/00171/1313.tif]

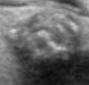

Supplement: Supplemental Information 2 — The raw data shows the demographics and clinicopathological features of all patient which were used to statistical analysis and built DL model. [file peerj-12-16952-s002.zip › Submited_Images/00172/11.tif]

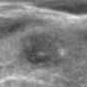

Supplement: Supplemental Information 2 — The raw data shows the demographics and clinicopathological features of all patient which were used to statistical analysis and built DL model. [file peerj-12-16952-s002.zip › Submited_Images/00173/1212.tif]

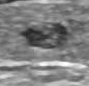

Supplement: Supplemental Information 2 — The raw data shows the demographics and clinicopathological features of all patient which were used to statistical analysis and built DL model. [file peerj-12-16952-s002.zip › Submited_Images/00175/11.tif]

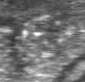

Supplement: Supplemental Information 2 — The raw data shows the demographics and clinicopathological features of all patient which were used to statistical analysis and built DL model. [file peerj-12-16952-s002.zip › Submited_Images/00178/33.tif]

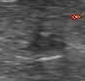

Supplement: Supplemental Information 2 — The raw data shows the demographics and clinicopathological features of all patient which were used to statistical analysis and built DL model. [file peerj-12-16952-s002.zip › Submited_Images/00181/22.tif]

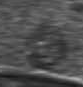

Supplement: Supplemental Information 2 — The raw data shows the demographics and clinicopathological features of all patient which were used to statistical analysis and built DL model. [file peerj-12-16952-s002.zip › Submited_Images/00183/33.tif]

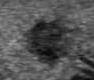

Supplement: Supplemental Information 2 — The raw data shows the demographics and clinicopathological features of all patient which were used to statistical analysis and built DL model. [file peerj-12-16952-s002.zip › Submited_Images/00184/22.tif]

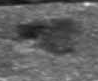

Supplement: Supplemental Information 2 — The raw data shows the demographics and clinicopathological features of all patient which were used to statistical analysis and built DL model. [file peerj-12-16952-s002.zip › Submited_Images/00188/33.tif]

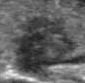

Supplement: Supplemental Information 2 — The raw data shows the demographics and clinicopathological features of all patient which were used to statistical analysis and built DL model. [file peerj-12-16952-s002.zip › Submited_Images/00189/22.tif]

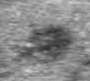

Supplement: Supplemental Information 2 — The raw data shows the demographics and clinicopathological features of all patient which were used to statistical analysis and built DL model. [file peerj-12-16952-s002.zip › Submited_Images/00190/66.tif]

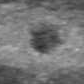

Supplement: Supplemental Information 2 — The raw data shows the demographics and clinicopathological features of all patient which were used to statistical analysis and built DL model. [file peerj-12-16952-s002.zip › Submited_Images/00192/33.tif]

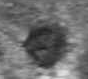

Supplement: Supplemental Information 2 — The raw data shows the demographics and clinicopathological features of all patient which were used to statistical analysis and built DL model. [file peerj-12-16952-s002.zip › Submited_Images/00193/22.tif]

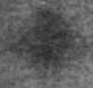

Supplement: Supplemental Information 2 — The raw data shows the demographics and clinicopathological features of all patient which were used to statistical analysis and built DL model. [file peerj-12-16952-s002.zip › Submited_Images/00196/11.tif]

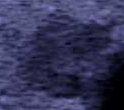

Supplement: Supplemental Information 2 — The raw data shows the demographics and clinicopathological features of all patient which were used to statistical analysis and built DL model. [file peerj-12-16952-s002.zip › Submited_Images/00198/11.tif]

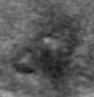

Supplement: Supplemental Information 2 — The raw data shows the demographics and clinicopathological features of all patient which were used to statistical analysis and built DL model. [file peerj-12-16952-s002.zip › Submited_Images/00199/11.tif]

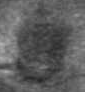

Supplement: Supplemental Information 2 — The raw data shows the demographics and clinicopathological features of all patient which were used to statistical analysis and built DL model. [file peerj-12-16952-s002.zip › Submited_Images/00201/22.tif]

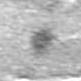

Supplement: Supplemental Information 2 — The raw data shows the demographics and clinicopathological features of all patient which were used to statistical analysis and built DL model. [file peerj-12-16952-s002.zip › Submited_Images/00202/33.tif]

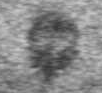

Supplement: Supplemental Information 2 — The raw data shows the demographics and clinicopathological features of all patient which were used to statistical analysis and built DL model. [file peerj-12-16952-s002.zip › Submited_Images/00203/11.tif]

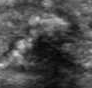

Supplement: Supplemental Information 2 — The raw data shows the demographics and clinicopathological features of all patient which were used to statistical analysis and built DL model. [file peerj-12-16952-s002.zip › Submited_Images/00204/22.tif]

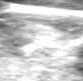

Supplement: Supplemental Information 2 — The raw data shows the demographics and clinicopathological features of all patient which were used to statistical analysis and built DL model. [file peerj-12-16952-s002.zip › Submited_Images/00206/33.tif]

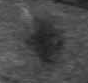

Supplement: Supplemental Information 2 — The raw data shows the demographics and clinicopathological features of all patient which were used to statistical analysis and built DL model. [file peerj-12-16952-s002.zip › Submited_Images/00207/44.tif]

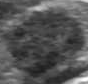

Supplement: Supplemental Information 2 — The raw data shows the demographics and clinicopathological features of all patient which were used to statistical analysis and built DL model. [file peerj-12-16952-s002.zip › Submited_Images/00208/22.tif]

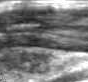

Supplement: Supplemental Information 2 — The raw data shows the demographics and clinicopathological features of all patient which were used to statistical analysis and built DL model. [file peerj-12-16952-s002.zip › Submited_Images/00209/66.tif]

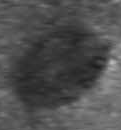

Supplement: Supplemental Information 2 — The raw data shows the demographics and clinicopathological features of all patient which were used to statistical analysis and built DL model. [file peerj-12-16952-s002.zip › Submited_Images/00213/55.tif]

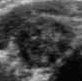

Supplement: Supplemental Information 2 — The raw data shows the demographics and clinicopathological features of all patient which were used to statistical analysis and built DL model. [file peerj-12-16952-s002.zip › Submited_Images/00215/11.tif]

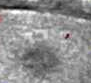

Supplement: Supplemental Information 2 — The raw data shows the demographics and clinicopathological features of all patient which were used to statistical analysis and built DL model. [file peerj-12-16952-s002.zip › Submited_Images/00219/22.tif]

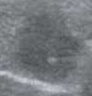

Supplement: Supplemental Information 2 — The raw data shows the demographics and clinicopathological features of all patient which were used to statistical analysis and built DL model. [file peerj-12-16952-s002.zip › Submited_Images/00222/11.tif]

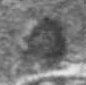

Supplement: Supplemental Information 2 — The raw data shows the demographics and clinicopathological features of all patient which were used to statistical analysis and built DL model. [file peerj-12-16952-s002.zip › Submited_Images/00223/33.tif]

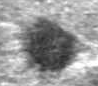

Supplement: Supplemental Information 2 — The raw data shows the demographics and clinicopathological features of all patient which were used to statistical analysis and built DL model. [file peerj-12-16952-s002.zip › Submited_Images/00224/11.tif]

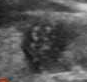

Supplement: Supplemental Information 2 — The raw data shows the demographics and clinicopathological features of all patient which were used to statistical analysis and built DL model. [file peerj-12-16952-s002.zip › Submited_Images/00225/11.tif]

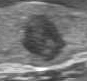

Supplement: Supplemental Information 2 — The raw data shows the demographics and clinicopathological features of all patient which were used to statistical analysis and built DL model. [file peerj-12-16952-s002.zip › Submited_Images/00226/33.tif]

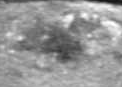

Supplement: Supplemental Information 2 — The raw data shows the demographics and clinicopathological features of all patient which were used to statistical analysis and built DL model. [file peerj-12-16952-s002.zip › Submited_Images/00227/22.tif]

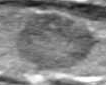

Supplement: Supplemental Information 2 — The raw data shows the demographics and clinicopathological features of all patient which were used to statistical analysis and built DL model. [file peerj-12-16952-s002.zip › Submited_Images/00229/11.tif]

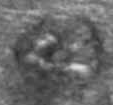

Supplement: Supplemental Information 2 — The raw data shows the demographics and clinicopathological features of all patient which were used to statistical analysis and built DL model. [file peerj-12-16952-s002.zip › Submited_Images/00232/11.tif]

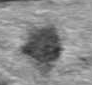

Supplement: Supplemental Information 2 — The raw data shows the demographics and clinicopathological features of all patient which were used to statistical analysis and built DL model. [file peerj-12-16952-s002.zip › Submited_Images/00233/33.tif]

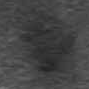

Supplement: Supplemental Information 2 — The raw data shows the demographics and clinicopathological features of all patient which were used to statistical analysis and built DL model. [file peerj-12-16952-s002.zip › Submited_Images/00236/11.tif]

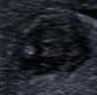

Supplement: Supplemental Information 2 — The raw data shows the demographics and clinicopathological features of all patient which were used to statistical analysis and built DL model. [file peerj-12-16952-s002.zip › Submited_Images/00237/22.tif]

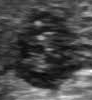

Supplement: Supplemental Information 2 — The raw data shows the demographics and clinicopathological features of all patient which were used to statistical analysis and built DL model. [file peerj-12-16952-s002.zip › Submited_Images/00238/11.tif]

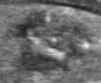

Supplement: Supplemental Information 2 — The raw data shows the demographics and clinicopathological features of all patient which were used to statistical analysis and built DL model. [file peerj-12-16952-s002.zip › Submited_Images/00239/22.tif]

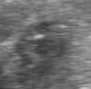

Supplement: Supplemental Information 2 — The raw data shows the demographics and clinicopathological features of all patient which were used to statistical analysis and built DL model. [file peerj-12-16952-s002.zip › Submited_Images/00240/11.tif]

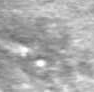

Supplement: Supplemental Information 2 — The raw data shows the demographics and clinicopathological features of all patient which were used to statistical analysis and built DL model. [file peerj-12-16952-s002.zip › Submited_Images/00241/22.tif]

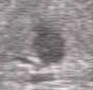

Supplement: Supplemental Information 2 — The raw data shows the demographics and clinicopathological features of all patient which were used to statistical analysis and built DL model. [file peerj-12-16952-s002.zip › Submited_Images/00243/22.tif]

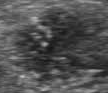

Supplement: Supplemental Information 2 — The raw data shows the demographics and clinicopathological features of all patient which were used to statistical analysis and built DL model. [file peerj-12-16952-s002.zip › Submited_Images/00244/11.tif]

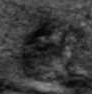

Supplement: Supplemental Information 2 — The raw data shows the demographics and clinicopathological features of all patient which were used to statistical analysis and built DL model. [file peerj-12-16952-s002.zip › Submited_Images/00245/11.tif]

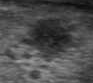

Supplement: Supplemental Information 2 — The raw data shows the demographics and clinicopathological features of all patient which were used to statistical analysis and built DL model. [file peerj-12-16952-s002.zip › Submited_Images/00246/11.tif]

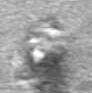

Supplement: Supplemental Information 2 — The raw data shows the demographics and clinicopathological features of all patient which were used to statistical analysis and built DL model. [file peerj-12-16952-s002.zip › Submited_Images/00247/22.tif]

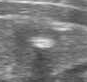

Supplement: Supplemental Information 2 — The raw data shows the demographics and clinicopathological features of all patient which were used to statistical analysis and built DL model. [file peerj-12-16952-s002.zip › Submited_Images/00248/11.tif]

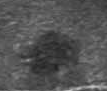

Supplement: Supplemental Information 2 — The raw data shows the demographics and clinicopathological features of all patient which were used to statistical analysis and built DL model. [file peerj-12-16952-s002.zip › Submited_Images/00250/33.tif]

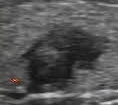

Supplement: Supplemental Information 2 — The raw data shows the demographics and clinicopathological features of all patient which were used to statistical analysis and built DL model. [file peerj-12-16952-s002.zip › Submited_Images/00251/11.tif]

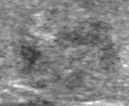

Supplement: Supplemental Information 2 — The raw data shows the demographics and clinicopathological features of all patient which were used to statistical analysis and built DL model. [file peerj-12-16952-s002.zip › Submited_Images/00252/22.tif]

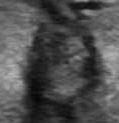

Supplement: Supplemental Information 2 — The raw data shows the demographics and clinicopathological features of all patient which were used to statistical analysis and built DL model. [file peerj-12-16952-s002.zip › Submited_Images/00255/22.tif]

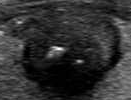

Supplement: Supplemental Information 2 — The raw data shows the demographics and clinicopathological features of all patient which were used to statistical analysis and built DL model. [file peerj-12-16952-s002.zip › Submited_Images/00256/77.tif]

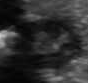

Supplement: Supplemental Information 2 — The raw data shows the demographics and clinicopathological features of all patient which were used to statistical analysis and built DL model. [file peerj-12-16952-s002.zip › Submited_Images/00257/33.tif]

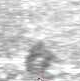

Supplement: Supplemental Information 2 — The raw data shows the demographics and clinicopathological features of all patient which were used to statistical analysis and built DL model. [file peerj-12-16952-s002.zip › Submited_Images/00259/22.tif]

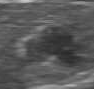

Supplement: Supplemental Information 2 — The raw data shows the demographics and clinicopathological features of all patient which were used to statistical analysis and built DL model. [file peerj-12-16952-s002.zip › Submited_Images/00260/11.tif]

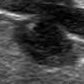

Supplement: Supplemental Information 2 — The raw data shows the demographics and clinicopathological features of all patient which were used to statistical analysis and built DL model. [file peerj-12-16952-s002.zip › Submited_Images/00261/88.tif]

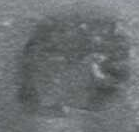

Supplement: Supplemental Information 2 — The raw data shows the demographics and clinicopathological features of all patient which were used to statistical analysis and built DL model. [file peerj-12-16952-s002.zip › Submited_Images/00262/22.tif]

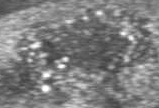

Supplement: Supplemental Information 2 — The raw data shows the demographics and clinicopathological features of all patient which were used to statistical analysis and built DL model. [file peerj-12-16952-s002.zip › Submited_Images/00263/22.tif]

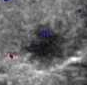

Supplement: Supplemental Information 2 — The raw data shows the demographics and clinicopathological features of all patient which were used to statistical analysis and built DL model. [file peerj-12-16952-s002.zip › Submited_Images/00264/11.tif]

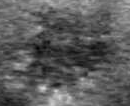

Supplement: Supplemental Information 2 — The raw data shows the demographics and clinicopathological features of all patient which were used to statistical analysis and built DL model. [file peerj-12-16952-s002.zip › Submited_Images/00265/11.tif]

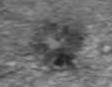

Supplement: Supplemental Information 2 — The raw data shows the demographics and clinicopathological features of all patient which were used to statistical analysis and built DL model. [file peerj-12-16952-s002.zip › Submited_Images/00266/11.tif]

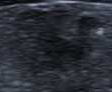

Supplement: Supplemental Information 2 — The raw data shows the demographics and clinicopathological features of all patient which were used to statistical analysis and built DL model. [file peerj-12-16952-s002.zip › Submited_Images/00267/33.tif]

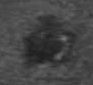

Supplement: Supplemental Information 2 — The raw data shows the demographics and clinicopathological features of all patient which were used to statistical analysis and built DL model. [file peerj-12-16952-s002.zip › Submited_Images/00270/11.tif]

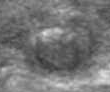

Supplement: Supplemental Information 2 — The raw data shows the demographics and clinicopathological features of all patient which were used to statistical analysis and built DL model. [file peerj-12-16952-s002.zip › Submited_Images/00272/33.tif]

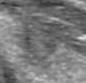

Supplement: Supplemental Information 2 — The raw data shows the demographics and clinicopathological features of all patient which were used to statistical analysis and built DL model. [file peerj-12-16952-s002.zip › Submited_Images/00273/33.tif]

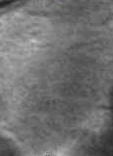

Supplement: Supplemental Information 2 — The raw data shows the demographics and clinicopathological features of all patient which were used to statistical analysis and built DL model. [file peerj-12-16952-s002.zip › Submited_Images/00275/11.tif]

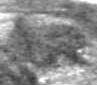

Supplement: Supplemental Information 2 — The raw data shows the demographics and clinicopathological features of all patient which were used to statistical analysis and built DL model. [file peerj-12-16952-s002.zip › Submited_Images/00276/11.tif]

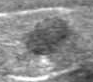

Supplement: Supplemental Information 2 — The raw data shows the demographics and clinicopathological features of all patient which were used to statistical analysis and built DL model. [file peerj-12-16952-s002.zip › Submited_Images/00278/22.tif]

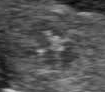

Supplement: Supplemental Information 2 — The raw data shows the demographics and clinicopathological features of all patient which were used to statistical analysis and built DL model. [file peerj-12-16952-s002.zip › Submited_Images/00279/66.tif]
