# Supplementary figures and images for: Predicting central cervical lymph node metastasis in papillary thyroid microcarcinoma using deep learning (part 1 of 2)
Source: PeerJ. 2024 Mar 29;12:e16952. doi: 10.7717/peerj.16952 (PMC10984175; doi:10.7717/peerj.16952)

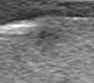

Supplement: Supplemental Information 2 — The raw data shows the demographics and clinicopathological features of all patient which were used to statistical analysis and built DL model. [file peerj-12-16952-s002.zip › Submited_Images/00001/66.tif]

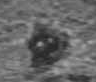

Supplement: Supplemental Information 2 — The raw data shows the demographics and clinicopathological features of all patient which were used to statistical analysis and built DL model. [file peerj-12-16952-s002.zip › Submited_Images/00002/11.tif]

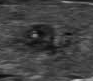

Supplement: Supplemental Information 2 — The raw data shows the demographics and clinicopathological features of all patient which were used to statistical analysis and built DL model. [file peerj-12-16952-s002.zip › Submited_Images/00004/44.tif]

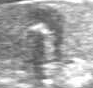

Supplement: Supplemental Information 2 — The raw data shows the demographics and clinicopathological features of all patient which were used to statistical analysis and built DL model. [file peerj-12-16952-s002.zip › Submited_Images/00005/11.tif]

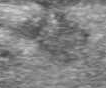

Supplement: Supplemental Information 2 — The raw data shows the demographics and clinicopathological features of all patient which were used to statistical analysis and built DL model. [file peerj-12-16952-s002.zip › Submited_Images/00006/22.tif]

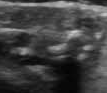

Supplement: Supplemental Information 2 — The raw data shows the demographics and clinicopathological features of all patient which were used to statistical analysis and built DL model. [file peerj-12-16952-s002.zip › Submited_Images/00007/55.tif]

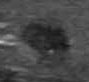

Supplement: Supplemental Information 2 — The raw data shows the demographics and clinicopathological features of all patient which were used to statistical analysis and built DL model. [file peerj-12-16952-s002.zip › Submited_Images/00008/33.tif]

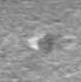

Supplement: Supplemental Information 2 — The raw data shows the demographics and clinicopathological features of all patient which were used to statistical analysis and built DL model. [file peerj-12-16952-s002.zip › Submited_Images/00009/44.tif]

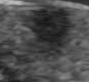

Supplement: Supplemental Information 2 — The raw data shows the demographics and clinicopathological features of all patient which were used to statistical analysis and built DL model. [file peerj-12-16952-s002.zip › Submited_Images/00010/22.tif]

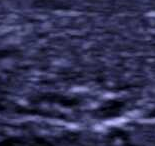

Supplement: Supplemental Information 2 — The raw data shows the demographics and clinicopathological features of all patient which were used to statistical analysis and built DL model. [file peerj-12-16952-s002.zip › Submited_Images/00011/77.tif]

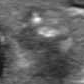

Supplement: Supplemental Information 2 — The raw data shows the demographics and clinicopathological features of all patient which were used to statistical analysis and built DL model. [file peerj-12-16952-s002.zip › Submited_Images/00014/33.tif]

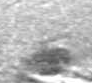

Supplement: Supplemental Information 2 — The raw data shows the demographics and clinicopathological features of all patient which were used to statistical analysis and built DL model. [file peerj-12-16952-s002.zip › Submited_Images/00016/1212.tif]

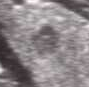

Supplement: Supplemental Information 2 — The raw data shows the demographics and clinicopathological features of all patient which were used to statistical analysis and built DL model. [file peerj-12-16952-s002.zip › Submited_Images/00017/44.tif]

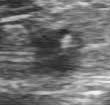

Supplement: Supplemental Information 2 — The raw data shows the demographics and clinicopathological features of all patient which were used to statistical analysis and built DL model. [file peerj-12-16952-s002.zip › Submited_Images/00019/333.tif]

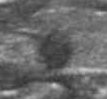

Supplement: Supplemental Information 2 — The raw data shows the demographics and clinicopathological features of all patient which were used to statistical analysis and built DL model. [file peerj-12-16952-s002.zip › Submited_Images/00020/11.tif]

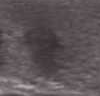

Supplement: Supplemental Information 2 — The raw data shows the demographics and clinicopathological features of all patient which were used to statistical analysis and built DL model. [file peerj-12-16952-s002.zip › Submited_Images/00022/111.tif]

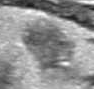

Supplement: Supplemental Information 2 — The raw data shows the demographics and clinicopathological features of all patient which were used to statistical analysis and built DL model. [file peerj-12-16952-s002.zip › Submited_Images/00023/333.tif]

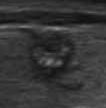

Supplement: Supplemental Information 2 — The raw data shows the demographics and clinicopathological features of all patient which were used to statistical analysis and built DL model. [file peerj-12-16952-s002.zip › Submited_Images/00024/111.jpg]

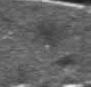

Supplement: Supplemental Information 2 — The raw data shows the demographics and clinicopathological features of all patient which were used to statistical analysis and built DL model. [file peerj-12-16952-s002.zip › Submited_Images/00026/222.tif]

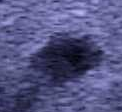

Supplement: Supplemental Information 2 — The raw data shows the demographics and clinicopathological features of all patient which were used to statistical analysis and built DL model. [file peerj-12-16952-s002.zip › Submited_Images/00027/222.tif]

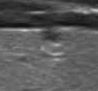

Supplement: Supplemental Information 2 — The raw data shows the demographics and clinicopathological features of all patient which were used to statistical analysis and built DL model. [file peerj-12-16952-s002.zip › Submited_Images/00030/777.tif]

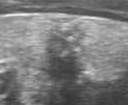

Supplement: Supplemental Information 2 — The raw data shows the demographics and clinicopathological features of all patient which were used to statistical analysis and built DL model. [file peerj-12-16952-s002.zip › Submited_Images/00031/555.tif]

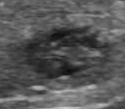

Supplement: Supplemental Information 2 — The raw data shows the demographics and clinicopathological features of all patient which were used to statistical analysis and built DL model. [file peerj-12-16952-s002.zip › Submited_Images/00032/11.tif]

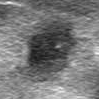

Supplement: Supplemental Information 2 — The raw data shows the demographics and clinicopathological features of all patient which were used to statistical analysis and built DL model. [file peerj-12-16952-s002.zip › Submited_Images/00033/66.tif]

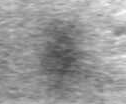

Supplement: Supplemental Information 2 — The raw data shows the demographics and clinicopathological features of all patient which were used to statistical analysis and built DL model. [file peerj-12-16952-s002.zip › Submited_Images/00034/33.tif]

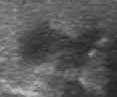

Supplement: Supplemental Information 2 — The raw data shows the demographics and clinicopathological features of all patient which were used to statistical analysis and built DL model. [file peerj-12-16952-s002.zip › Submited_Images/00035/666.tif]

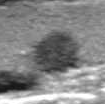

Supplement: Supplemental Information 2 — The raw data shows the demographics and clinicopathological features of all patient which were used to statistical analysis and built DL model. [file peerj-12-16952-s002.zip › Submited_Images/00036/222.tif]

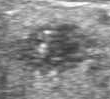

Supplement: Supplemental Information 2 — The raw data shows the demographics and clinicopathological features of all patient which were used to statistical analysis and built DL model. [file peerj-12-16952-s002.zip › Submited_Images/00038/111.tif]

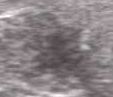

Supplement: Supplemental Information 2 — The raw data shows the demographics and clinicopathological features of all patient which were used to statistical analysis and built DL model. [file peerj-12-16952-s002.zip › Submited_Images/00039/111.tif]

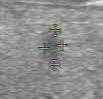

Supplement: Supplemental Information 2 — The raw data shows the demographics and clinicopathological features of all patient which were used to statistical analysis and built DL model. [file peerj-12-16952-s002.zip › Submited_Images/00040/333.tif]

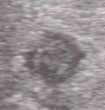

Supplement: Supplemental Information 2 — The raw data shows the demographics and clinicopathological features of all patient which were used to statistical analysis and built DL model. [file peerj-12-16952-s002.zip › Submited_Images/00041/55.tif]

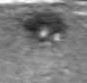

Supplement: Supplemental Information 2 — The raw data shows the demographics and clinicopathological features of all patient which were used to statistical analysis and built DL model. [file peerj-12-16952-s002.zip › Submited_Images/00042/222.tif]

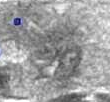

Supplement: Supplemental Information 2 — The raw data shows the demographics and clinicopathological features of all patient which were used to statistical analysis and built DL model. [file peerj-12-16952-s002.zip › Submited_Images/00044/222.tif]

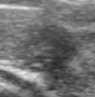

Supplement: Supplemental Information 2 — The raw data shows the demographics and clinicopathological features of all patient which were used to statistical analysis and built DL model. [file peerj-12-16952-s002.zip › Submited_Images/00045/111.tif]

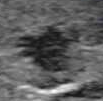

Supplement: Supplemental Information 2 — The raw data shows the demographics and clinicopathological features of all patient which were used to statistical analysis and built DL model. [file peerj-12-16952-s002.zip › Submited_Images/00046/111.tif]

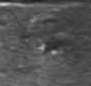

Supplement: Supplemental Information 2 — The raw data shows the demographics and clinicopathological features of all patient which were used to statistical analysis and built DL model. [file peerj-12-16952-s002.zip › Submited_Images/00048/222.tif]

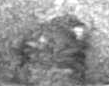

Supplement: Supplemental Information 2 — The raw data shows the demographics and clinicopathological features of all patient which were used to statistical analysis and built DL model. [file peerj-12-16952-s002.zip › Submited_Images/00049/111.tif]

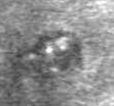

Supplement: Supplemental Information 2 — The raw data shows the demographics and clinicopathological features of all patient which were used to statistical analysis and built DL model. [file peerj-12-16952-s002.zip › Submited_Images/00050/222.tif]

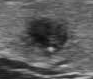

Supplement: Supplemental Information 2 — The raw data shows the demographics and clinicopathological features of all patient which were used to statistical analysis and built DL model. [file peerj-12-16952-s002.zip › Submited_Images/00051/44.tif]

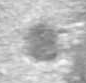

Supplement: Supplemental Information 2 — The raw data shows the demographics and clinicopathological features of all patient which were used to statistical analysis and built DL model. [file peerj-12-16952-s002.zip › Submited_Images/00052/444.tif]

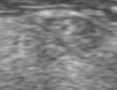

Supplement: Supplemental Information 2 — The raw data shows the demographics and clinicopathological features of all patient which were used to statistical analysis and built DL model. [file peerj-12-16952-s002.zip › Submited_Images/00053/888.tif]

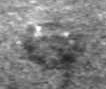

Supplement: Supplemental Information 2 — The raw data shows the demographics and clinicopathological features of all patient which were used to statistical analysis and built DL model. [file peerj-12-16952-s002.zip › Submited_Images/00055/222.tif]

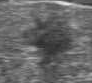

Supplement: Supplemental Information 2 — The raw data shows the demographics and clinicopathological features of all patient which were used to statistical analysis and built DL model. [file peerj-12-16952-s002.zip › Submited_Images/00056/22.tif]

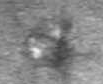

Supplement: Supplemental Information 2 — The raw data shows the demographics and clinicopathological features of all patient which were used to statistical analysis and built DL model. [file peerj-12-16952-s002.zip › Submited_Images/00057/11.tif]

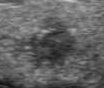

Supplement: Supplemental Information 2 — The raw data shows the demographics and clinicopathological features of all patient which were used to statistical analysis and built DL model. [file peerj-12-16952-s002.zip › Submited_Images/00058/444.tif]

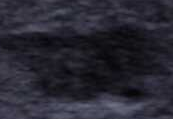

Supplement: Supplemental Information 2 — The raw data shows the demographics and clinicopathological features of all patient which were used to statistical analysis and built DL model. [file peerj-12-16952-s002.zip › Submited_Images/00059/222.tif]

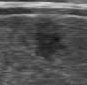

Supplement: Supplemental Information 2 — The raw data shows the demographics and clinicopathological features of all patient which were used to statistical analysis and built DL model. [file peerj-12-16952-s002.zip › Submited_Images/00060/111.tif]

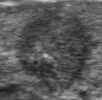

Supplement: Supplemental Information 2 — The raw data shows the demographics and clinicopathological features of all patient which were used to statistical analysis and built DL model. [file peerj-12-16952-s002.zip › Submited_Images/00061/222.tif]

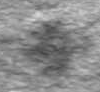

Supplement: Supplemental Information 2 — The raw data shows the demographics and clinicopathological features of all patient which were used to statistical analysis and built DL model. [file peerj-12-16952-s002.zip › Submited_Images/00062/44.tif]

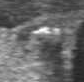

Supplement: Supplemental Information 2 — The raw data shows the demographics and clinicopathological features of all patient which were used to statistical analysis and built DL model. [file peerj-12-16952-s002.zip › Submited_Images/00063/66.tif]

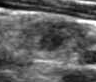

Supplement: Supplemental Information 2 — The raw data shows the demographics and clinicopathological features of all patient which were used to statistical analysis and built DL model. [file peerj-12-16952-s002.zip › Submited_Images/00064/888.tif]

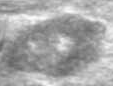

Supplement: Supplemental Information 2 — The raw data shows the demographics and clinicopathological features of all patient which were used to statistical analysis and built DL model. [file peerj-12-16952-s002.zip › Submited_Images/00065/111.tif]

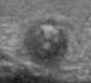

Supplement: Supplemental Information 2 — The raw data shows the demographics and clinicopathological features of all patient which were used to statistical analysis and built DL model. [file peerj-12-16952-s002.zip › Submited_Images/00066/333.tif]

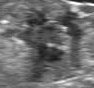

Supplement: Supplemental Information 2 — The raw data shows the demographics and clinicopathological features of all patient which were used to statistical analysis and built DL model. [file peerj-12-16952-s002.zip › Submited_Images/00067/111.tif]

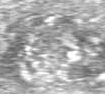

Supplement: Supplemental Information 2 — The raw data shows the demographics and clinicopathological features of all patient which were used to statistical analysis and built DL model. [file peerj-12-16952-s002.zip › Submited_Images/00068/11.tif]

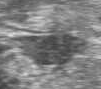

Supplement: Supplemental Information 2 — The raw data shows the demographics and clinicopathological features of all patient which were used to statistical analysis and built DL model. [file peerj-12-16952-s002.zip › Submited_Images/00069/22.tif]

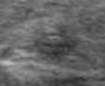

Supplement: Supplemental Information 2 — The raw data shows the demographics and clinicopathological features of all patient which were used to statistical analysis and built DL model. [file peerj-12-16952-s002.zip › Submited_Images/00072/44.tif]

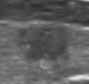

Supplement: Supplemental Information 2 — The raw data shows the demographics and clinicopathological features of all patient which were used to statistical analysis and built DL model. [file peerj-12-16952-s002.zip › Submited_Images/00074/11.tif]

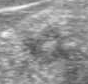

Supplement: Supplemental Information 2 — The raw data shows the demographics and clinicopathological features of all patient which were used to statistical analysis and built DL model. [file peerj-12-16952-s002.zip › Submited_Images/00075/55.tif]

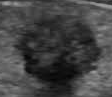

Supplement: Supplemental Information 2 — The raw data shows the demographics and clinicopathological features of all patient which were used to statistical analysis and built DL model. [file peerj-12-16952-s002.zip › Submited_Images/00077/11.tif]

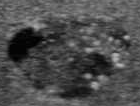

Supplement: Supplemental Information 2 — The raw data shows the demographics and clinicopathological features of all patient which were used to statistical analysis and built DL model. [file peerj-12-16952-s002.zip › Submited_Images/00079/33.tif]

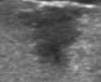

Supplement: Supplemental Information 2 — The raw data shows the demographics and clinicopathological features of all patient which were used to statistical analysis and built DL model. [file peerj-12-16952-s002.zip › Submited_Images/00080/55.tif]

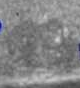

Supplement: Supplemental Information 2 — The raw data shows the demographics and clinicopathological features of all patient which were used to statistical analysis and built DL model. [file peerj-12-16952-s002.zip › Submited_Images/00082/11.tif]

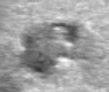

Supplement: Supplemental Information 2 — The raw data shows the demographics and clinicopathological features of all patient which were used to statistical analysis and built DL model. [file peerj-12-16952-s002.zip › Submited_Images/00084/22.tif]

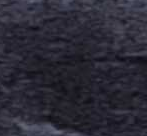

Supplement: Supplemental Information 2 — The raw data shows the demographics and clinicopathological features of all patient which were used to statistical analysis and built DL model. [file peerj-12-16952-s002.zip › Submited_Images/00086/77.tif]

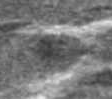

Supplement: Supplemental Information 2 — The raw data shows the demographics and clinicopathological features of all patient which were used to statistical analysis and built DL model. [file peerj-12-16952-s002.zip › Submited_Images/00087/33.tif]

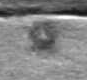

Supplement: Supplemental Information 2 — The raw data shows the demographics and clinicopathological features of all patient which were used to statistical analysis and built DL model. [file peerj-12-16952-s002.zip › Submited_Images/00088/11.tif]

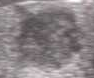

Supplement: Supplemental Information 2 — The raw data shows the demographics and clinicopathological features of all patient which were used to statistical analysis and built DL model. [file peerj-12-16952-s002.zip › Submited_Images/00089/11.tif]

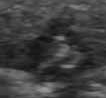

Supplement: Supplemental Information 2 — The raw data shows the demographics and clinicopathological features of all patient which were used to statistical analysis and built DL model. [file peerj-12-16952-s002.zip › Submited_Images/00090/22.tif]

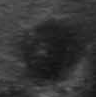

Supplement: Supplemental Information 2 — The raw data shows the demographics and clinicopathological features of all patient which were used to statistical analysis and built DL model. [file peerj-12-16952-s002.zip › Submited_Images/00092/33.tif]

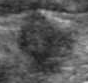

Supplement: Supplemental Information 2 — The raw data shows the demographics and clinicopathological features of all patient which were used to statistical analysis and built DL model. [file peerj-12-16952-s002.zip › Submited_Images/00094/44.tif]

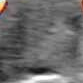

Supplement: Supplemental Information 2 — The raw data shows the demographics and clinicopathological features of all patient which were used to statistical analysis and built DL model. [file peerj-12-16952-s002.zip › Submited_Images/00097/33.tif]

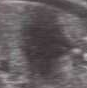

Supplement: Supplemental Information 2 — The raw data shows the demographics and clinicopathological features of all patient which were used to statistical analysis and built DL model. [file peerj-12-16952-s002.zip › Submited_Images/00098/55.tif]

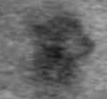

Supplement: Supplemental Information 2 — The raw data shows the demographics and clinicopathological features of all patient which were used to statistical analysis and built DL model. [file peerj-12-16952-s002.zip › Submited_Images/00100/11.tif]

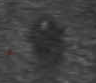

Supplement: Supplemental Information 2 — The raw data shows the demographics and clinicopathological features of all patient which were used to statistical analysis and built DL model. [file peerj-12-16952-s002.zip › Submited_Images/00101/1818.tif]

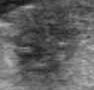

Supplement: Supplemental Information 2 — The raw data shows the demographics and clinicopathological features of all patient which were used to statistical analysis and built DL model. [file peerj-12-16952-s002.zip › Submited_Images/00103/22.tif]

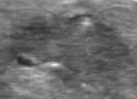

Supplement: Supplemental Information 2 — The raw data shows the demographics and clinicopathological features of all patient which were used to statistical analysis and built DL model. [file peerj-12-16952-s002.zip › Submited_Images/00104/22.tif]

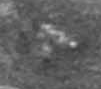

Supplement: Supplemental Information 2 — The raw data shows the demographics and clinicopathological features of all patient which were used to statistical analysis and built DL model. [file peerj-12-16952-s002.zip › Submited_Images/00105/44.tif]

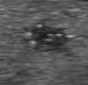

Supplement: Supplemental Information 2 — The raw data shows the demographics and clinicopathological features of all patient which were used to statistical analysis and built DL model. [file peerj-12-16952-s002.zip › Submited_Images/00106/22.tif]

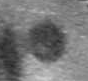

Supplement: Supplemental Information 2 — The raw data shows the demographics and clinicopathological features of all patient which were used to statistical analysis and built DL model. [file peerj-12-16952-s002.zip › Submited_Images/00107/88.tif]

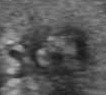

Supplement: Supplemental Information 2 — The raw data shows the demographics and clinicopathological features of all patient which were used to statistical analysis and built DL model. [file peerj-12-16952-s002.zip › Submited_Images/00108/55.tif]

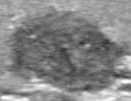

Supplement: Supplemental Information 2 — The raw data shows the demographics and clinicopathological features of all patient which were used to statistical analysis and built DL model. [file peerj-12-16952-s002.zip › Submited_Images/00109/77.tif]

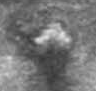

Supplement: Supplemental Information 2 — The raw data shows the demographics and clinicopathological features of all patient which were used to statistical analysis and built DL model. [file peerj-12-16952-s002.zip › Submited_Images/00110/33.tif]

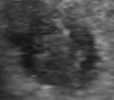

Supplement: Supplemental Information 2 — The raw data shows the demographics and clinicopathological features of all patient which were used to statistical analysis and built DL model. [file peerj-12-16952-s002.zip › Submited_Images/00111/44.tif]

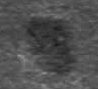

Supplement: Supplemental Information 2 — The raw data shows the demographics and clinicopathological features of all patient which were used to statistical analysis and built DL model. [file peerj-12-16952-s002.zip › Submited_Images/00112/11.tif]

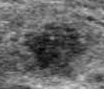

Supplement: Supplemental Information 2 — The raw data shows the demographics and clinicopathological features of all patient which were used to statistical analysis and built DL model. [file peerj-12-16952-s002.zip › Submited_Images/00113/22.tif]

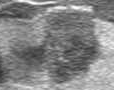

Supplement: Supplemental Information 2 — The raw data shows the demographics and clinicopathological features of all patient which were used to statistical analysis and built DL model. [file peerj-12-16952-s002.zip › Submited_Images/00114/77.tif]

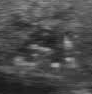

Supplement: Supplemental Information 2 — The raw data shows the demographics and clinicopathological features of all patient which were used to statistical analysis and built DL model. [file peerj-12-16952-s002.zip › Submited_Images/00115/44.tif]

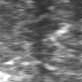

Supplement: Supplemental Information 2 — The raw data shows the demographics and clinicopathological features of all patient which were used to statistical analysis and built DL model. [file peerj-12-16952-s002.zip › Submited_Images/00116/44.tif]

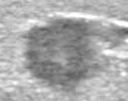

Supplement: Supplemental Information 2 — The raw data shows the demographics and clinicopathological features of all patient which were used to statistical analysis and built DL model. [file peerj-12-16952-s002.zip › Submited_Images/00117/44.tif]

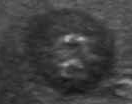

Supplement: Supplemental Information 2 — The raw data shows the demographics and clinicopathological features of all patient which were used to statistical analysis and built DL model. [file peerj-12-16952-s002.zip › Submited_Images/00119/44.tif]

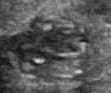

Supplement: Supplemental Information 2 — The raw data shows the demographics and clinicopathological features of all patient which were used to statistical analysis and built DL model. [file peerj-12-16952-s002.zip › Submited_Images/00120/22.tif]

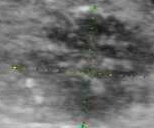

Supplement: Supplemental Information 2 — The raw data shows the demographics and clinicopathological features of all patient which were used to statistical analysis and built DL model. [file peerj-12-16952-s002.zip › Submited_Images/00121/77.tif]

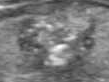

Supplement: Supplemental Information 2 — The raw data shows the demographics and clinicopathological features of all patient which were used to statistical analysis and built DL model. [file peerj-12-16952-s002.zip › Submited_Images/00122/33.tif]

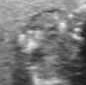

Supplement: Supplemental Information 2 — The raw data shows the demographics and clinicopathological features of all patient which were used to statistical analysis and built DL model. [file peerj-12-16952-s002.zip › Submited_Images/00124/33.tif]

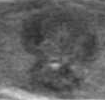

Supplement: Supplemental Information 2 — The raw data shows the demographics and clinicopathological features of all patient which were used to statistical analysis and built DL model. [file peerj-12-16952-s002.zip › Submited_Images/00125/22.tif]

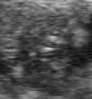

Supplement: Supplemental Information 2 — The raw data shows the demographics and clinicopathological features of all patient which were used to statistical analysis and built DL model. [file peerj-12-16952-s002.zip › Submited_Images/00127/44.tif]

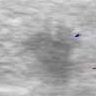

Supplement: Supplemental Information 2 — The raw data shows the demographics and clinicopathological features of all patient which were used to statistical analysis and built DL model. [file peerj-12-16952-s002.zip › Submited_Images/00128/22.tif]

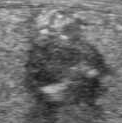

Supplement: Supplemental Information 2 — The raw data shows the demographics and clinicopathological features of all patient which were used to statistical analysis and built DL model. [file peerj-12-16952-s002.zip › Submited_Images/00129/77.tif]

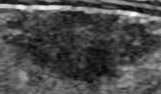

Supplement: Supplemental Information 2 — The raw data shows the demographics and clinicopathological features of all patient which were used to statistical analysis and built DL model. [file peerj-12-16952-s002.zip › Submited_Images/00130/77.tif]
